# Effect of an aqueous 4% titanium tetrafluoride solution on preventing caries in orthodontic patients: a controlled clinical trial

**DOI:** 10.1590/2177-6709.28.1.e2321304.oar

**Published:** 2023-04-03

**Authors:** Ana Beatriz CHEVITARESE, Karla Lorene de França LEITE, Paulo Bechara DUTRA, Matheus Melo PITHON, Lucianne Cople Maia FARIA, Antônio Carlos de Oliveira RUELLAS

**Affiliations:** 1 Universidade Federal do Rio de Janeiro, Departamento de Ortodontia e Odontologia Pediátrica (Rio de Janeiro/RJ, Brazil).; 2 Instituto de Química Analítica da Universidade Federal do Rio de Janeiro (Rio do Janeiro/RJ, Brazil).; 3 Universidade do Sudeste da Bahia, Departamento de Ortodontia (Jequié/BA, Brazil).

**Keywords:** Dental caries, Dental enamel, Fluorides

## Abstract

**Introduction:**

Titanium tetrafluoride (TiF_4_) is a fluoride compound that, when is applied over enamel, promotes a protection against demineralization through a titanium dioxide (TiO_2_) acid-resistant coat.

**Objectives:**

This study sought to verify the hypothesis that a single application of 4% TiF_4_ increases the resistance of enamel to dental demineralization in orthodontic patients.

**Materials and Methods:**

This controlled clinical trial followed CONSORT guidelines and investigated the prevention of enamel demineralization, fluoride retention, and the presence of a Ti layer after TiF_4_ application on banded teeth exposed to clinical cariogenic biofilm. Forty premolars were divided into a control group (CG; n = 20) and a test group (TG; n = 20). Teeth from both groups received prophylaxis and orthodontic bands with a cariogenic locus. In the TG, all teeth additionally underwent aqueous 4% TiF_4_ solution application after prophylaxis before being banded. After one month, teeth from both groups were extracted and prepared to assess the microhardness, fluoride retention, and evaluation of the Ti coating over the enamel surface. All data were analyzed with a paired Student’s t-test (p<0.05).

**Results:**

Enamel microhardness and fluoride uptake were higher in TG than in CG, while the Ti layer could be seen over TG teeth that received TiF_4_ application.

**Conclusion:**

Under clinical circumstances, the 4% aqueous TiF_4_ solution was effective in preventing enamel mineral loss through increasing the enamel resistance to dental demineralization, enhancing its microhardness and fluoride uptake, and forming a Ti coat.

## RESUMO

**Introdução:**

O tetrafluoreto de titânio (TiF_4_) é um composto fluoretado que, quando aplicado sobre o esmalte dentário, promove uma proteção contra desmineralização, por meio da formação de uma camada ácido-resistente de dióxido de titânio (TiO_2_).

**Objetivos:**

O presente estudo buscou verificar a hipótese de que uma única aplicação de TiF_4_ a 4% aumenta a resistência do esmalte dentário à desmineralização, em pacientes ortodônticos.

**Material e Métodos:**

Esse ensaio clínico controlado seguiu as diretrizes do CONSORT e investigou a prevenção da desmineralização do esmalte, retenção de flúor e a presença de uma camada de titânio após a aplicação do TiF_4_ em dentes bandados expostos ao biofilme cariogênico clínico. Quarenta pré-molares foram divididos em dois grupos: controle (GC; n = 20) e teste (GT; n = 20). Dentes de ambos os grupos receberam profilaxia e bandas ortodônticas com nicho cariogênico. No GT, todos os dentes também foram submetidos a aplicação de solução aquosa de TiF_4_ 4 após profilaxia, antes de serem bandados. Após um mês, os dentes de ambos os grupos foram extraídos e preparados para avaliar a microdureza, retenção de flúor e avaliação da camada de titânio sobre a superfície do esmalte. Todos os dados foram analisados pelo teste *t* pareado (p<0,05).

**Resultados:**

A microdureza do esmalte e a absorção de flúor foram mais elevadas no GT do que no GC, enquanto uma camada de titânio pôde ser observada sobre os dentes do GT, que receberam aplicação TiF_4_.

**Conclusão:**

Em circunstâncias clínicas, a solução de TiF_4_ a 4% foi eficaz na prevenção da perda mineral do esmalte, por meio do aumento da resistência à desmineralização dentária, aumentando sua microdureza e absorção de flúor, e formando uma camada protetora de titânio.

## INTRODUCTION

Orthodontic treatment with fixed appliances has been established as an integral part of contemporary orthodontics due to its capacity for precisely planned tooth movements in all three planes of space.^1^ However, it is well-known that orthodontic patients are more inclined to develop dental caries as a result of their greater difficulty in maintaining adequate plaque control by tooth-brushing, flossing, and mouth-rinsing with fluorides during years of treatment,^2^ ^,^ ^3^ creating a favorable condition for plaque to rapidly adhere and accumulate.^4^ ^,^ ^5^ As a result, enamel demineralization and gingivitis have come to be regarded as the most prevalent consequences of biofilm formation, affecting 50% to 70% of patients with fixed appliances.^6^

Fluorides are used in Dentistry to improve the resistance of human enamel against acid attack^1^. However, most of the success of fluoride therapy is patient-dependent, often requiring consecutive appointments to become effective.

Titanium tetrafluoride (TiF_4_) began to be researched in 1972 by Mundorff et al.^7^ and Shrestha et al.^8^, who described its capacity to cover the tooth surface and ability to promote higher rates of fluoride penetration and retention in the enamel. The created layer also demonstrates less solubility under cariogenic challenge.

This Ti-rich coat, formed by titanium dioxide (TiO_2_), is more resistant than any other fluoride agent,^7^ decreasing enamel solubility and enamel porosity,^8^ reducing caries formation, and enhancing enamel fluoride concentration.^9^ Besides, it cannot be removed from the enamel surface by potassium hydroxide^10^ or with hydrogen chloride.^11^ According to Buyukyilmaz et al.^10^ the presence of this Ti coat could be verified over enamel up to one year after its application. 

Although there are many studies evaluating TiF_4_ efficacy in caries prevention,^12^ ^,^ ^13^ there are also some doubts regarding its effects in orthodontic patients. As such, this study sought to determine the microhardness and fluoride uptake and to evaluate the Ti coat over enamel after the application of 4% TiF_4_ on banded teeth submitted to a high *in vivo* cariogenic challenge. The hypothesis that a single application of this compound enhances enamel resistance was also investigated.

## MATERIAL AND METHODS

### TRIAL DESIGN

This non-randomized, controlled, split-mouth and single-blind clinical trial was developed and conducted involving patients of orthodontic clinics at an university in Rio de Janeiro (Brazil) and was approved by the research ethics committee. The patients signed a written consent before their participation on the trial.

### SAMPLE SIZE AND ELIGIBILITY CRITERIA

Ten orthodontic patients scheduled for extraction of the four first premolars (n=40) as part of their treatment plan were included. To be included, patients were required to be in good health, not be taking any medications, and not receiving any other medical treatment. Patients with teeth showing enamel defects or caries lesions (white spots or cavitation) observed under clinical analysis were excluded from the present study. The sample size calculation was based on the reduction in the enamel mineral loss in the 1% TiF_4_ solution group, compared to negative control (no treatment) observed in a previous study by Büyükyilmaz et al.^14^ Considering a power = 0.8, α = 0.05, and based on a two-sided test, a sample size of 15 blocks allocated into each group of treatment was required to complete the study. With 30% added to compensate for possible losses, at least 20 blocks for each group should be selected (BioEstat^®^ v.5.3, *Instituto de Desenvolvimento Sustentável Mamirauá*, Tefé, Brazil). The participants were blinded during all clinical procedures.

All clinical examinations and procedures were done by a non-single-blind calibrated operator at the orthodontic clinic of *Universidade Federal do Rio de Janeiro* (UFRJ, Rio de Janeiro, Brazil). The clinical procedures were done at the same appointment and patient’s instructions were given at the end of it.

### EXPERIMENTAL TIF_4_

The experimental 4% TiF_4_ solution was formulated by dissolving 3.4 g of TiF_4_ (Sigma-Aldrich Co., St. Louis, MO, USA) in 100 mL of deionized distilled water.^13^ The pH of the fresh solution was 1.0. 

### CLINICAL PROCEDURES INTERVENTIONS

All 40 premolars received prophylaxis (with fluoride-free prophylactic paste) and were divided (split-mouth) into two groups containing 20 teeth each: a test group (TG) containing teeth from the first and third quadrants, and a control group (CG) containing teeth from the second and fourth quadrants. This quadrant stratification arrangement was made to avoid any interference regarding which side of the mouth the patient brushes their teeth best. 

The CG teeth received prophylaxis and orthodontic bands with a cariogenic locus. The TG teeth, after receiving prophylaxis, also had their buccal surfaces treated by a direct passive application of 4% TiF_4_ solution (pH: 1.0) for 60 seconds with a Microbrush^®^ (KG Sorensen), followed by 60 seconds of water-rinsing and 10 seconds of compressed air-drying. These procedures were completed while avoiding any surface contamination by oral fluids, using cotton rolls and saliva suction. Thereafter, the TG teeth also received orthodontic bands with a cariogenic locus.

### CARIOGENIC LOCUS PREPARATION

**Figure 1:**
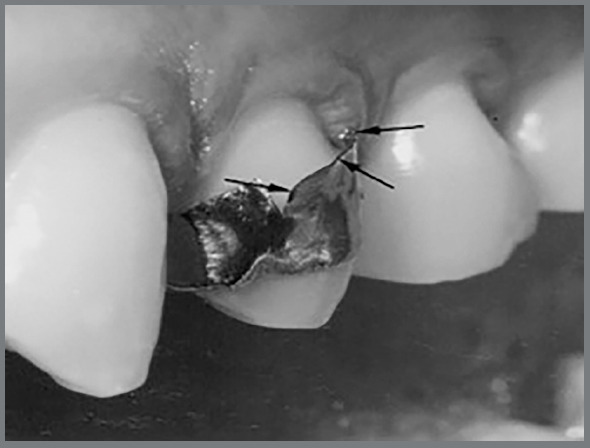
Experimental cariogenic locus.

The orthodontic bands received two cuts on the buccal surface, measuring 2 mm in length and separated 4 mm from each other, to create a retention area for biofilm formation (Fig 1). This area represented the experimental cariogenic locus in both the CG and TG.

Afterward, all bands were seated, completely adapted, and cemented with glass ionomer (Meron^®^; Voco, Cuxhaven, Germany) on the included premolars of both groups. These procedures were done by only one not-blinded calibrated orthodontist. Patients were instructed to maintain their normal diet and oral hygiene during the experimental period.

### LABORATORY PROCEDURES

After one month, all premolars were extracted by one blinded calibrated dentist, avoiding any damage to the premolar crowns, and the teeth were stored in a 0.1% solution of deionized water with thymol crystals (pH 7.0).^15^ After this point, the bands were carefully removed and all teeth were washed with deionized water to remove plaque and debris.

After root separation, each crown was mesiodistally cut under irrigation using a diamond disc and only buccal surfaces were prepared for laboratory analysis. Each half was included in epoxy resin except for a window (2 mm × 4 mm) on the buccal surface, which was the same as that exposed on the cariogenic locus. 

### ENAMEL MICROHARDNESS

**Figure 2:**
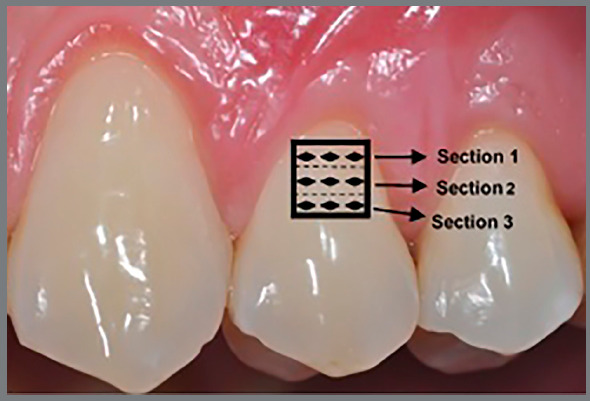
Sections at cariogenic locus.

Twenty teeth were prepared to be analyzed, by a blinded operator, for microhardness^12^. The indentations were made on the samples by Leitz load-hardness testing (Micromet 2003, model no. 16005300; Buehler Ltd., Lake Bluff, IL, USA) with a load of 25 g/5 s. The load transmission was always perpendicular to the enamel surface, and measurements were performed on three sections (Fig 2): section 1 (close to the cervical area), section 2 (50 µm occlusal from section 1), and section 3 (50 µm occlusal from section 2). Three indentations were performed for each section and the means of the three measurements were calculated.

### FLUORIDE UPTAKE

Each sample was individually weighed, immersed lifted by a nylon wire and stored in individual plastic containers with 10 mL of 1 M solution of potassium hydroxide (Merck reagent P.A.; Merck, Kenilworth, NJ, USA) under constant shaking for one week. After this period, each sample was weighed again to determine the fluoride uptake for each specimen. 

The analysis of fluoride ions was conducted by using 0.5 mL of the stored solutions. The free fluoride determination was measured using ion chromatography (Dionex DX-100; Thermo Fisher Scientific, Waltham, MA, USA) with suppressed conductivity. The instrument was fitted with an IonPac AS10 analytical column (Thermo Fisher Scientific, Waltham, MA, USA). Each stored solution was injected onto the injection loop of the instrument and a flow rate of 1.0 mL/min was adopted. Free fluoride ions have a well-defined retention time and the peak corresponding to fluoride can be readily determined from the chromatogram. The peak area was used to determine fluoride concentrations by linear interpolation between standard solution concentrations that were slightly higher and lower than that of the test solution^16^. All these fluoride analysis were done by a blinded operator.

### ENAMEL TI LAYER

One blinded operator covered 20 specimens with a carbon layer to be observed by scanning electron microscopy (model no. JSM 6460LV; JEOL Ltd., Tokyo, Japan). For each tooth, electromicrography was performed, and Ti mapping through the microprobe analysis with an energy dispersive spectrometer was completed (Noran System SIX, model no. 200; Thermo Fisher Scientific, Waltham, MA, USA) to determine the Ti coat presence.

### STATISTICAL METHODS

The obtained results of microhardness and fluoride retention were tabulated in Excel (Microsoft Corp., Redmond, WA, USA) and imported into the Statistical Package for the Social Sciences software program (IBM Corp., Armonk, NY, USA). Data distribution and normality were checked using the Shapiro-Wilk test. Differences between the control and test groups were statistically evaluated using a Student’s t-paired test with a significance level of 5% (p<0.05). The descriptive analysis was done considering whether or not the Ti coat showed through during microprobe analysis with an energy dispersive spectrometer, and the nature of its formation over human enamel.

## RESULTS

### PARTICIPANT FLOW

**Figure 3:**
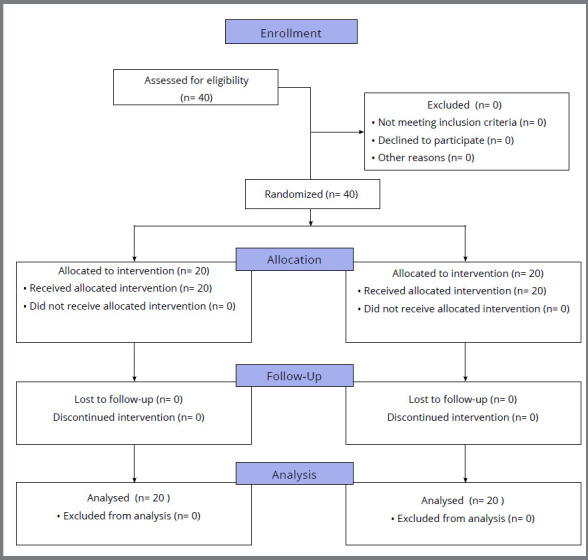
Participant flow.

All recruited participants remained at the end of the experiment, totalizing 40 teeth (20 of control group and 20 of test group) (Fig 3). The trial period was 30 days, starting with bands bonding and finishing at teeth extraction.

### ENAMEL MICROHARDNESS

The results of microhardness are compiled in Table 1, which lists the mean and standard deviation values of Knoop microhardness (Kgf/mm^2^) for the CG and TG.

**Table 1:** Microhardness (Kgf/mm^2^) values of control group (CG) and test group (TG) (average of three indentations).

Microhardness	n	Average	SD
Section 1 (TG)	20	345.41^A^	76.90
Section 1 (CG)	20	209.23^B^	54.57
Section 2 (TG)	20	341.14^A^	79.84
Section 2 (CG)	20	207.41^B^	57.72
Section 3 (TG)	20	332.63^A^	78.34
Section 3 (CG)	20	217.25^B^	51.02

Different uppercase letters in the same column show differences between the treatments (paired *t*-test p<0.05).

Statistical analysis revealed a significant difference between the CG (no treatment) and TG (TiF_4_ treatment) (p<0.05) after the *in vivo* experiment, indicating that microhardness was higher in the TG than in the CG. There were no differences between sections for the same experimental group (p>0.05).

### FLUORIDE UPTAKE

The mean values of fluoride uptake of the TG were higher than those of the CG in all specimens (n=40) of both quadrants (Table 2). 

**Table 2:** Fluoride uptake from test and control groups (mg/ml).

PATIENT	1^sd^ Quadrant	2^nd^ Quadrant	3^rd^ Quadrant	4^th^ Quadrant
	TG	CG	TG	CG
1	0.0056	0.0037	0.0059	0.0026
2	0.0063	0.0026	0.0071	0.0032
3	0.0057	0.0041	0.0062	0.0036
4	0.0048	0.0035	0.0054	0.0029
5	0.0046	0.0022	0.0057	0.0017
6	0.0077	0.0049	0.0082	0.0059
7	0.0065	0.0050	0.0069	0.0043
8	0.0052	0.0031	0.0070	0.0045
9	0.0075	0.0058	0.0081	0.0062
10	0.0044	0.0027	0.0037	0.0021

### ENAMEL TI LAYER

**Figure 4:**
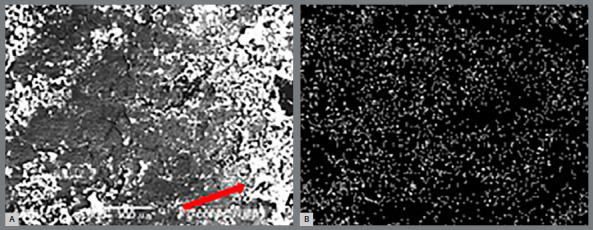
A) Enamel electro-micrography of TG teeth showing titanium layer. B) Titanium coat irregular formation.

**Figure 5:**
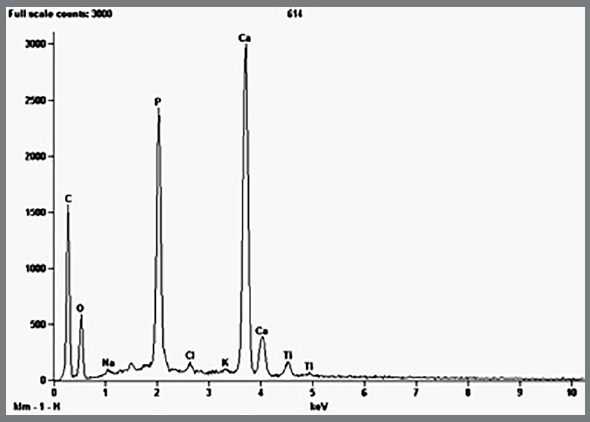
Titanium peak during mapping (TG).

The superficial Ti layer was present in 100% of the TG (n=20). The presence of Ti was established by electromicrography (Fig 4A) and it was verified that the Ti layer was irregular along the surface (Fig 4B). Ti mapping displayed a Ti peak graph (Fig 5).

**Figure 6:**
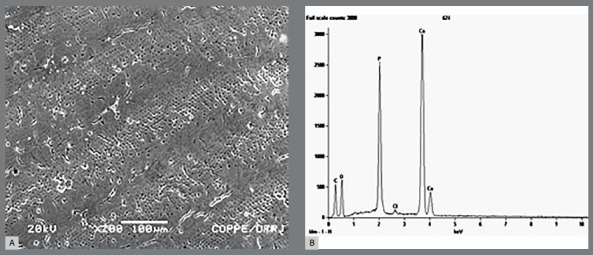
A) Enamel electromicrograph of CG tooth, showing sound enamel. B) Titanium peak absence during mapping (CG).

**Figure 7:**
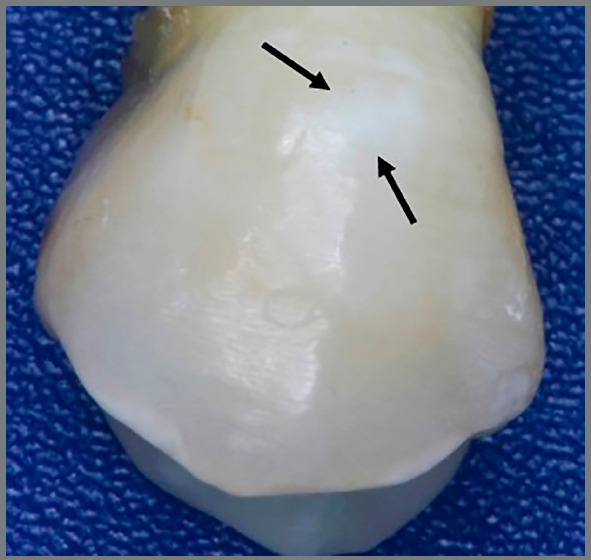
Demineralization areas.

In CG samples (n=20), the enamel appeared normal (Fig 6A) or showed demineralization in some areas (Fig 7), and no traces of Ti were detected by any Ti peak graph (Fig 6B).

## DISCUSSION

Although this non-randomized, controlled, split-mouth and single-blind clinical trial followed CONSORT guidelines and was conducted in a orthodontic clinic with a convenience sample, we believe that this clinical issue (caries below orthodontic bands) is a very common and recurrent problem in orthodontic treatment, faced by all orthodontists. 

The maintenance of enamel’s integrity after orthodontic treatment is one of the orthodontist’s primary goals. However, maintaining oral hygiene is often difficult and may be complicated by the presence of bands and brackets. Thus, the introduction of a product that enhances enamel’s resistance is very important for the orthodontic community. In this regard, the present study sought to determine the microhardness and the fluoride retention and to evaluate the Ti coat over enamel after the application of 4% TiF_4_ on banded teeth. 

Dental caries typically starts at and below the enamel surface, and are the result of a process in which the crystalline mineral structure of the tooth is demineralized by organic acids.^17^ ^,^ ^18^ The loss of tooth minerals leads to increased porosity, widening of the spaces between the enamel crystals, and softening of the tooth’s surface, which allows acids to diffuse increasingly deeper into the tooth, resulting in further demineralization below the surface (subsurface demineralization).^1^ ^,^ ^17^ However, the presence of fluoride can inhibit the demineralization of the surface layer, thus also protecting deeper structures as well.^19^

Although there are many methods to determine the demineralization process on human enamel, in this research, the *in vivo* model was chosen to develop dental demineralization, because *in vitro* tests have significant limitations, particularly including the incapacity to reproduce the biological processes that encompass the development of dental caries.^20^ In the present study, the same protocol of Buyukyilmaz et al^14^ was used, with a four-week experimental time to produce *in vivo* caries.

Microhardness measurement is one possible way of gathering information on the degree of demineralization or remineralization.^21^ The Knoop microhardness technique is the most commonly used approach and is a simple way of deriving hardness information on enamel.^21^ The adoption of this method, if applied perpendicular to the enamel surface, provides information at different depths of a caries lesion.^21^ In this study, the probe was applied as perpendicularly as possible to the enamel surface, right on the cervical area of the first premolar crown. Unfortunately, the premolar surface could not be flattened before the experiment because the teeth were in the patients’ mouths at the time, and after their extraction they were already covered by the aforementioned TiO_2_ layer.

The results obtained in this study for the TG (pretreated with TiF_4_) were more favorable in terms of avoiding demineralization, compared to those of the CG. Noteworthy, section 1 was the most susceptible area, due to its proximity to gingival tissues and to the edge of the band, which is more susceptible to showing margins that are not well-adapted. 

Reintsema and Arends^22^ observed a similar result regarding microhardness in human enamel. The present findings showed that the application of TiF_4_ not only protected the enamel, but also increased its resistance to caries. In fact, the CG experienced less enamel microhardness and showcased white spot formation in many samples.

Usually, fluoride concentration is higher on the enamel surface, decreasing slowly toward the enamel-dentin junction, where it increases again.^23^ The present trial revealed greater fluoride uptake occurred in the TG, compared to the CG. This fact agrees with many other studies that reported higher levels of fluoride present in enamel after the application of TiF_4_, when compared with acidulated phosphate fluoride (APF) or sodium fluoride.^7^ ^,^ ^24^ ^-^ ^27^

Gu et al.^23^ previously found that a slight application of TiF_4_ led to high concentrations of fluoride and titanium that remained for up to 22 weeks after treatment. Similarly, Skartveit et al.^28^ reported that a brief application of TiF_4_ resulted in the same degree of fluoride increase as four minutes of stannous fluoride (SnF_2_) application, although the enamel demineralization was smaller in the TiF_4_ group. Data from the present trial corroborate both of these studies’ results, showing that a single application of TiF_4_ could increase enamel fluoride uptake and protect the tooth surface against demineralization. According to Clarkson and Wefel,^24^ pretreatment with Ti ion over the enamel surface before fluoride application seems to be effective in increasing the anticaries potential. 

Although orthodontists provide hygiene instructions and dietary counseling, many patients are not able to maintain an adequate level of buccal hygiene, and may develop an ideal environment for the progression of demineralization and dental caries. 

The presence of caries lesions (from white spots to cavitation) underneath and around the edges of the bands is a common problem faced during orthodontic treatment. With the purpose of minimizing this problem, many agents for bonding and cementing have been produced with fluoride, such as glass ionomer.^29^ Nevertheless, even these fluoride-rich cements can be dissolved by oral fluids if the bands are not well adapted, creating an excellent cariogenic locus wherein the patient cannot clean with brushing or flossing. 

Although the TiO_2_ layer formation after the *in vitro* application of TiF_4_ over enamel surface can interfere in the adhesion mechanism, by decreasing the microshear bond strength of composite resin to enamel,^35^ as regards to orthodontic bands bonding, the authors of the present study believe that this TiF_4_ layer may not be a problem, since the use of TiF_4_ did not influence other bonding processes, like of fiber posts.^36^

In this study, 4% TiF_4_ was applied over TG tooth surfaces before banding, in an attempt to protect the enamel surfaces against cariogenic challenge. Van der Linden and Dermaut^30^ suggested that orthodontic bands (around their margins or underneath them) may facilitate enamel demineralization due to the lack of adaptation and cement dissolution brought on by oral fluids.

In CG teeth, it could be observed that some samples exhibited demineralized areas on their buccal surfaces, even though the enamel was in contact with the glass ionomer cement, which releases fluoride. None of the TG teeth showed signs of demineralization, perhaps because of their contact with TiF_4_ before bonding. It seems that, after the application of TiF_4_, the enamel was saturated with free fluoride ions liberated after the Ti ions broke the union with fluoride, which immediately linked to oxygen present in the enamel, forming a TiO_2_ coat^27^ over the enamel. The enamel can uptake this free fluoride that links to its calcium, forming calcium fluoride. Alexandria et al.^31^ also observed the TiF_4_ potential in preventing enamel demineralization under severe cariogenic challenge, and concluded that the TiO_2_ layer formed was effective on enamel protection.

Several authors have described the presence of a Ti-rich coat over human enamel after the application of TiF_4_.^8^ ^,^ ^10^ ^,^ ^11^ ^,^ ^25^ ^,^ ^27^ When TiF_4_ comes into contact with the dental structure, titanium breaks its union with fluoride and quickly links to oxygen on the enamel surface, forming a superficial coat of TiO_2_, which is chemically nontoxic in biological systems.^30^ ^,^ ^32^ The reaction is completed in seconds and both the titanium and fluoride ions seem to be involved in this process.^2^

According to the present findings, the presence of a titanium layer could be noted in 100% of the teeth from the TG, reinforcing the idea that, due to titanium’s attraction to the oxygen, TiO_2_ is formed before free titanium penetrates into the enamel.^33^ This enamel protection was effective for all TG samples, although the titanium layer was not homogeneous, which is expected when considering the enamel porosity and surface irregularity. 

According to Buyukyilmaz et al.^10^, the TiO_2_ coating could be seen after one year of its application over enamel surface. Besides, if this layer may remain over an occlusal surface for one year, when compared to a surface covered by orthodontic bands, a long-lasting protective effect against enamel surface demineralization will be present.

These findings encourage the performance of other studies exploring the clinical use of TiF_4_, including its staying time on teeth and reapplication needs. This material may be capable of protecting banded teeth against caries, by increasing enamel microhardness and fluoride uptake. 

## CONCLUSIONS

After the completion of this clinical controlled trial, it could be concluded that:

» Enamel microhardness was higher when enamel was exposed to 4% TiF_4_ before orthodontic band bonding, indicating that TiF_4_ has a good potential to prevent enamel demineralization.
» Fluoride retention was higher in teeth that received a single application of 4% TiF_4_ before orthodontic band placement.
» Formation of a rich coating occurred over the enamel surface after the application of 4% TiF_4_. Although the Ti layer was irregular, it was enough to keep the enamel surface caries-free during the four-week experimental period.
